# Pulmonary Embolism of COVID-19: A Year of Reflection

**DOI:** 10.7759/cureus.40638

**Published:** 2023-06-19

**Authors:** Margarida Agudo, Pedro Moura, Francisca Santos, Carolina Robalo, Adriano Carvalho, Sónia Serra

**Affiliations:** 1 Internal Medicine, Centro Hospitalar de Setúbal, Setúbal, PRT; 2 Medicine, Centro Hospitalar do Médio Ave, Vila Nova de Famalicão, PRT; 3 Radiology, Instituto Português de Oncologia do Porto, Porto, PRT

**Keywords:** pulmonary embolism, pulmonary embolism predisposing factor, immunothrombosis, pulmonary embolism (pe), covid-19 complication, covid-19

## Abstract

Introduction

The coronavirus disease 2019 (COVID-19) pandemic has brought about significant changes in the medical field. While primarily characterized as a respiratory syndrome, COVID-19 is also associated with vascular events, particularly thrombotic complications. These events can manifest as initial presentations or develop as complications during the course of the disease, predominantly driven by immune-mediated mechanisms.

Methods

Patients with thrombotic complications followed in the post-COVID-19 thrombosis consult of 2021 were retrospectively analyzed and assessed for predisposing factors for pulmonary embolism (PE), including thrombophilias. Patients underwent reassessments over a minimum six-month period following diagnosis to evaluate vascular reperfusion and the potential discontinuation of anticoagulant therapy.

Results

All patients with PE exhibited segmental or subsegmental PE. Pulmonary CT angiography revealed that only one patient did not show complete reperfusion after six months of anticoagulant therapy alone. There were no instances of recurrent thrombotic events observed during this observation period. Among the studied patients, hypertension, diabetes, and obesity were identified as the most prevalent predisposing factors. No patients were diagnosed with thrombophilias or other relevant factors. Despite extensive research on the predisposing mechanisms of this complication in recent years, limited data exist regarding patients with this specific complication.

Discussion and conclusion

Continued research into COVID-19 patients and their complications is crucial for understanding the pathophysiological mechanisms and risk factors associated with these complications. The findings of this study support the existence of a multifactorial mechanism, with a significant pro-inflammatory component exacerbated by pre-existing risk factors, rather than a purely prothrombotic mechanism.

## Introduction

The coronavirus disease 2019 (COVID-19) virus was first reported in December 2019 in the city of Wuhan, China, and reached levels of spread that led the World Health Organization (WHO) to declare a pandemic in March 2020. This virus has a varied spectrum of manifestations, ranging from asymptomatic cases to critical illness, which can result in death. While respiratory symptoms are the predominant presentation, vascular events and complications are also observed, with thrombotic pathology being a major factor [1-3]. Among these, pulmonary embolism (PE) holds a prominent position, primarily affecting males with an average age of 60 years [4,5]. Similar to non-COVID-19-related PE, Virchow's triad, consisting of endothelial damage, stasis, and a hypercoagulable state, is involved in its pathophysiology. However, the occurrence of this triad is not attributed to precipitating factors or genetic/acquired coagulation defects.

In this case, PE occurs due to a state of immunologically mediated hyperinflammation, with several dysregulated immune factors, giving rise to the concept of immunothrombosis. This process inhibits fibrinolysis, leading to increased fibrin production and subsequent degradation, resulting in elevated levels of D-dimer. Thus, D-dimer measurement has become an excellent marker of inflammation and the likelihood of thrombotic events [3-9]. Moreover, this condition primarily affects pneumocytes due to the virus's high affinity for alveolar tissue, particularly the microvasculature [6].

As a result, the majority of thrombotic events manifest as pulmonary thromboembolism, mainly in a segmental and subsegmental distribution [10,11]. Chest angiotomography (angio-CT) is the preferred imaging exam for mapping these thrombotic events [10,11]. Regarding treatment, it is similar to thromboembolism of other etiologies, requiring the initiation of anticoagulation for a recommended duration of three to six months.

Given the considerable number of patients with COVID-19-associated thrombotic complications, we decided to monitor and further study this subgroup of patients, particularly during the post-acute phase of these vascular events. Consequently, a post-COVID-19 follow-up medical appointment was established for patients who experienced such complications during COVID-19. This article focuses on the analysis of patients followed in this medical appointment throughout the year 2021.

## Materials and methods

This study involved a review of clinical processes and imaging examinations for patients who were followed up in the post-COVID-19 thrombosis consultation and had experienced PE by a retrospective analysis of patients' medical records between January 2021 and December 2021. All patients were included in the study and there were no exclusion criteria. The primary objectives were the evaluation of the progression of thrombosis in these patients utilizing clinical and imaging modalities and identifying any potential associations between risk factors and the occurrence of these events in this specific population. A descriptive study was conducted using SPSS (IBM Corp., Armonk, NY) and Microsoft Excel (Microsoft Corporation, Redmond, WA) for data analysis.

## Results

**Figure 1:**
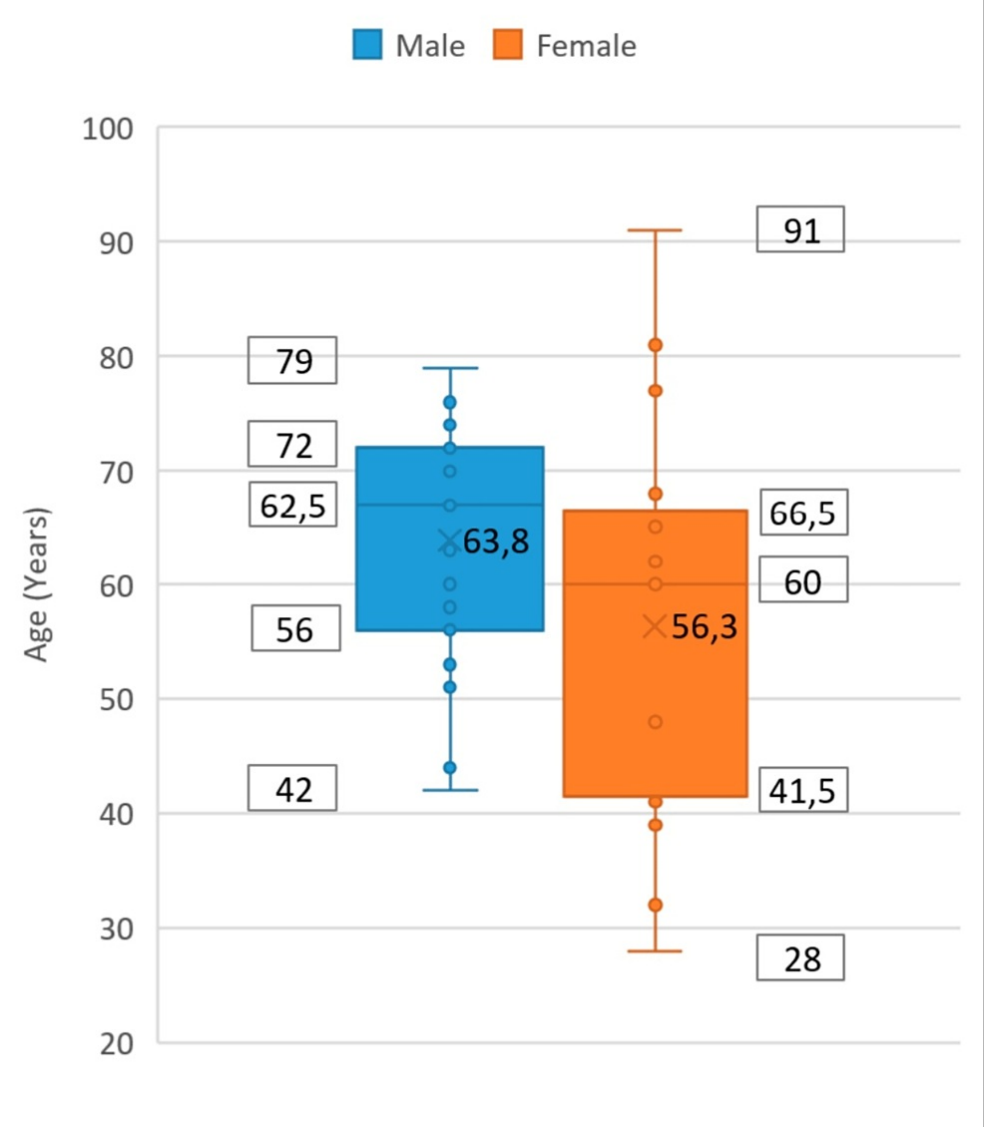
Age distribution associated with gender in patients with pulmonary embolism

After careful analysis of all patients followed in the post-COVID thrombosis consultation, a sample of 50 patients was obtained. Approximately 54% of the patients were male and 46% were female, who presented a minimum and maximum age of 28 and 91 years, respectively, with a median value of 60.62, as can be seen in Figure 1.

**Figure 2:**
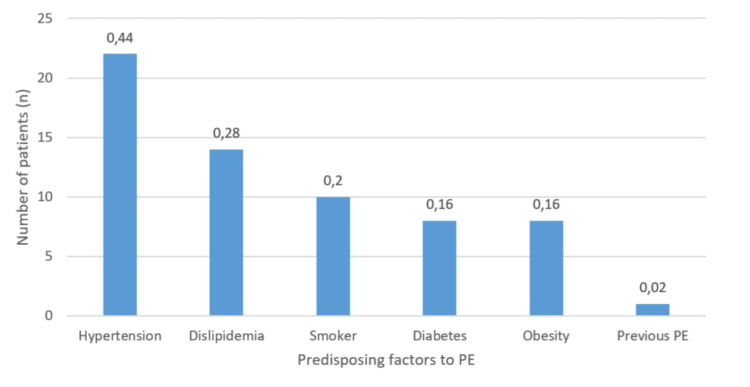
Number of patients with predisposing factors to pulmonary embolism (PE)

Regarding comorbidities, patients had significant cardiovascular risk factors (CVRF), contributing to an inflammatory state, and consequently increasing the likelihood of thrombotic events associated with COVID-19. Hypertension was the most prevalent, affecting approximately 44% of the patients, followed by dyslipidemia in 28% of the patients. Non-insulin-treated diabetes mellitus and obesity ranked third and fourth, respectively, affecting approximately 16% of the patients. It is also known that 20% of these patients were smokers, which also contributes to a pro-inflammatory state. However, the smoking burden is unknown. Figure 2 illustrates these findings. None of the patients had previous thrombophilias (genetic or acquired), and only 2% had a previous history of PE.

**Figure 3:**
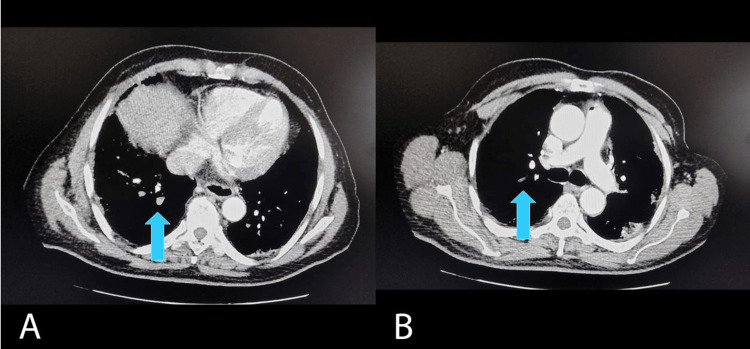
Images A and B show angio-CT scans with segmental and subsegmental pulmonary embolisms due to COVID-19, respectively

Regarding the thrombotic event, 90% of the patients had PE associated with COVID-19. Among these, 35.6% had a subsegmental location on chest angio-CT scans, while 31.1% had a segmental location, and both locations were present in 33.3% of the sample. Figure 3 shows an example of an angio-CT scan with the mapping of these thrombotic events in these preferential locations.

**Figure 4:**
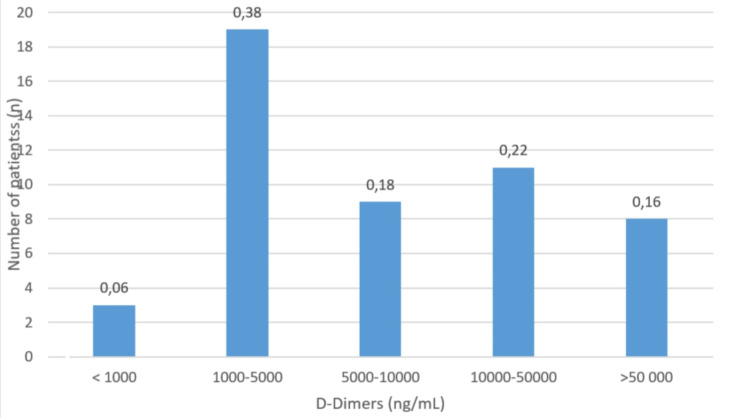
Distribution of D-dimer levels (Ng/ml: nanograms per milliliter) in patients with pulmonary embolism due to COVID-19

The analytical assessment of inflammation was based on the quantification of D-dimers during the acute phase of the thrombotic event. During this phase, it was found that 38% of the patients had D-dimer levels between 1000 and 5000 nanograms per milliliter (ng/mL; reference value < 500 ng/mL), while 22% had levels between 10,000 and 50,000 D-dimers. Furthermore, 18% had levels between 5,000 and 10,000 D-dimers, and 16% of the patients had levels exceeding 50,000 D-dimers. Figure 4 shows this distribution.

None of the patients showed alterations in coagulation times, including thrombin time and activated partial thromboplastin time (aPTT). All patients were treated with anticoagulation using novel oral anticoagulants (NOACs): apixaban, edoxaban, and rivaroxaban at full doses for three to six months. During treatment with anticoagulation, no adverse effects and/or signs of bleeding dyscrasia were observed. At the end of this period, subsequent laboratory and imaging reassessments were made.

**Figure 5:**
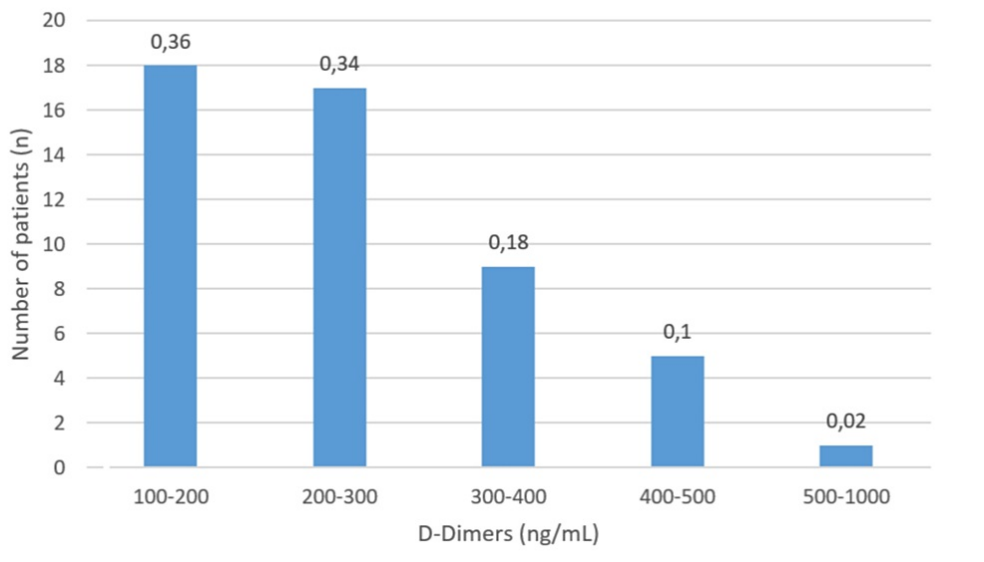
Distribution of D-dimer levels (Ng/ml: nanograms per milliliter) in patients with pulmonary embolism after six months of anticoagulation treatment

After six months, it was found that 36% of the patients had D-dimer levels ranging between 100 and 200 ng/mL, while 34% had measurements between 200 and 300 ng/mL. Only 2% of the patients had persistent measurements exceeding 500 ng/mL. This distribution is shown in Figure 5.

Regarding the imaging evaluation using CT angiography (CTA), after six months, only one patient presented areas of chronic hypoperfusion and hypoventilation. In the remaining patients, CTA confirmed complete repermeabilization of the vascular system.

In light of these findings, anticoagulation therapy was discontinued, and the patients were kept under surveillance for another month. None of the patients experienced a recurrence of the thrombotic event.

## Discussion

The COVID-19 pandemic has completely changed the paradigm of medicine as we know it. We were confronted with an unknown virus with initially unpredictable behavior, which made research and understanding of it challenging. Despite all the adversities it imposed, it has been possible to investigate and deepen our knowledge, improving the treatment and prognosis of patients. In this sense, this descriptive analysis was conducted to corroborate and reinforce what is described in the literature.

Compared to thrombotic events not caused by this virus, there is a common denominator that equally contributes to the development of thromboembolism: obesity. In thromboembolism not associated with COVID-19, obesity is a risk factor by promoting stasis and hypercoagulability of Virchow's triad. However, in thrombotic events associated with COVID-19, obesity contributes to immunothrombosis by acting as a pro-inflammatory factor [11]. Similarly, the presence of other comorbidities, such as diabetes, dyslipidemia, and hypertension, seem to increase inflammation and contribute to this immune-mediated state [5].

In COVID-19-associated PE, it is observed that the virus causes endothelial injury leading to the activation of interferon and other pro-inflammatory mediators, which in turn induce an immune-mediated response with macrophage activation syndrome, resulting in interstitial and endothelial inflammation. On the other hand, endothelial injury activates mediators such as kallikrein and angiotensin 2, causing vascular damage and consequent vasodilation, contributing to impaired fibrinolysis and coagulopathy. Consequently, there is a decrease in nitric oxide production and an increase in fibrin production, among other mediators, leading to thrombosis [2,6].

Overall, this analysis highlights the complex interplay between COVID-19 infection, inflammation, endothelial dysfunction, and thrombotic events, shedding light on the pathophysiological mechanisms underlying thromboembolic complications associated with the disease.

**Figure 6:**
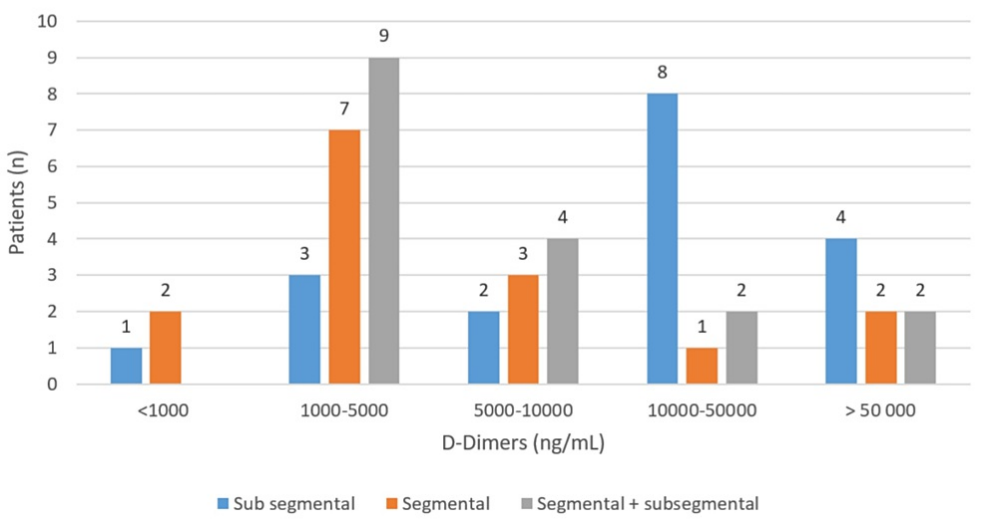
Correlation between diagnostic D-dimers and location of pulmonary embolism

In contrast, in thrombotic events not associated with COVID-19, except for idiopathic cases (rare), the genesis of the thrombus is mediated by alterations in the coagulation cascade caused by a coagulation deficiency or an acquired risk factor. Thus, the state of hypercoagulability is enhanced, leading to the occurrence of the thrombotic event. On the contrary, in thrombotic events associated with COVID-19, there are no alterations in the coagulation factors or the presence of thrombophilia in PE. Supporting these findings, coagulation times mostly remain unchanged, while D-dimer levels, reflecting the existing inflammatory state, are elevated. It was also observed that patients with more comorbidities presented higher D-dimer levels, further confirming this pro-inflammatory state that enhances thrombosis. However, the extent or location of thromboembolism on imaging exams does not seem to be correlated with the levels of these markers. Figure 6 illustrates the lack of correlation between D-dimers and the location of thrombotic events.

According to the literature, the extent of the inflammation and the occurrence of thrombosis can also be assessed by measuring fibrinogen and immune mediators such as interferon-alpha and interleukins [4-11]. However, due to the chaotic situation and lack of resources at times, these measurements were unfortunately not performed or available, which could have contributed to reinforcing this theory.

It is also known that COVID-19 has a high affinity for angiotensin II receptors, which are predominantly found in lung tissue, particularly in the alveolar endothelium [4,6]. Thus it was found that the majority of thrombotic events are pulmonary, which corroborates the tropism of this virus. In comparison to PE due to other causes, deep venous thrombosis is usually associated, which is not observed in thrombotic events due to COVID-19 (PE and deep venous thrombosis are isolated events). As for the distribution in the pulmonary territory, thrombotic events are mostly segmental and/or subsegmental, further supporting the affinity of immunothrombosis for the microvasculature, as described in the literature [6,8,9].

**Figure 7:**
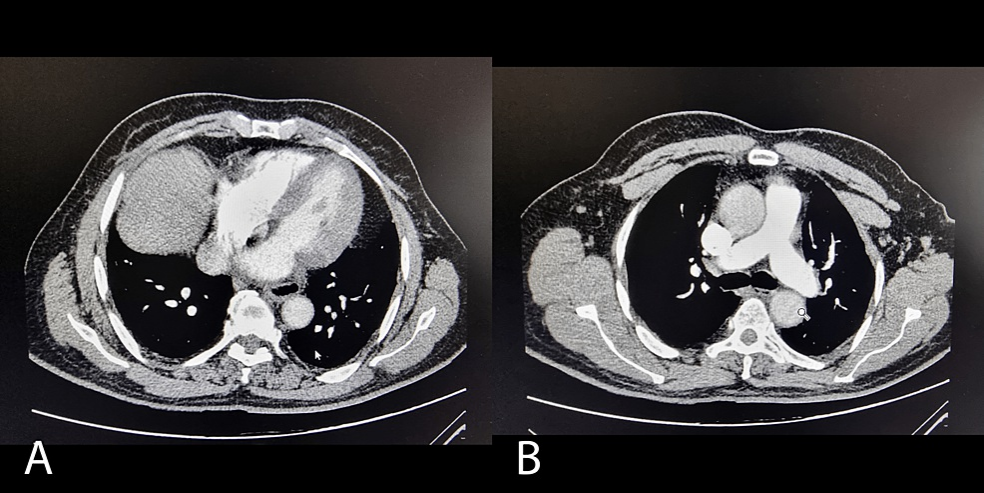
Images A and B show angio-CT scans with repermeabilization of a pulmonary embolism due to COVID-19 (after six months), respectively

Although anticoagulation treatment is similar for both types of thromboembolisms (COVID-19 associated vs. PE due to other causes), its effect is more pronounced and persistent in thrombotic events associated with COVID-19. After six months, 98% of patients had D-dimer levels below the clinical significance threshold (less than 200 ng/mL) and total repermeabilization of pulmonary vessels. Only 2% of patients had D-dimer levels above 500 ng/mL and imaging features consistent with chronic filling defects. In contrast, this outcome is not observed in thrombotic events not associated with COVID-19. In these cases, despite strict adherence to anticoagulation and suspension of predisposing risk factors, a longer time window is required for the resolution of the thrombotic event, and many patients end up developing chronic thromboembolism. Based on these findings, the therapeutic efficacy and favorable prognosis in COVID-19-associated PE may be due to the immune-mediated nature of thrombotic events. Thus, with the resolution of the viral condition, the thrombogenic mechanisms cease, leading to total repermeabilization of the vascular system and the possibility of anticoagulation discontinuation. Figure 7 illustrates this recovery process on imaging.

These patients were subsequently followed up, and no recurrence of thrombotic events was observed, with the patients remaining clinically asymptomatic and stable. This is not always the case with non-COVID-associated thromboembolism, as recurrence of thrombotic events is observed. When there is a thrombophilia or a persistent risk factor, the relapse of the thrombotic event is usually within the first weeks after discontinuing therapy. Therefore, despite COVID-19 having an aggressive course with severe complications, it presents a better prognosis compared to thrombotic events of other etiologies.

## Conclusions

The emergence of the COVID-19 virus in December 2019 in Wuhan, China, resulted in a global pandemic by March 2020. This virus exhibits a diverse range of manifestations, with thrombotic events being observed. PE plays a significant role in these thrombotic events, predominantly affecting older males. The pathophysiology of COVID-19-associated PE involves immunologically mediated hyperinflammation, leading to a state of immunothrombosis. This process hampers fibrinolysis, leading to increased fibrin production and elevated levels of D-dimers, which serve as markers of inflammation and thrombotic risk. Most thrombotic events manifest as pulmonary thromboembolism, primarily in the segmental and subsegmental regions of the lungs. Chest angiotomography is the preferred imaging technique for mapping these events, and treatment involves anticoagulation for a recommended period of three to six months.

The analysis of this study underscores the intricate interplay between COVID-19 infection, inflammation, endothelial dysfunction, and thrombotic events. COVID-19-associated thrombosis differs from non-COVID-19-related thrombosis in terms of its underlying mechanisms and treatment response. The immunothrombotic nature of COVID-19-associated thrombosis may contribute to a more favorable prognosis and a lower risk of recurrence compared to thrombotic events caused by other factors. Continued research and understanding of the pathophysiological mechanisms driving COVID-19-associated thrombotic events are crucial for enhancing treatment and prognosis for affected individuals. Despite the challenges presented by the pandemic, efforts to investigate and expand knowledge have resulted in advancements in medical practices. This descriptive analysis adds to the existing literature, reinforcing the significance of comprehensive management of thrombotic complications in COVID-19 patients.
